# HIV Pre-Exposure Prophylaxis Use Among South Asian Sexual Minority Men in the United States: Pilot Web-Based Survey Study

**DOI:** 10.2196/88920

**Published:** 2026-08-11

**Authors:** Akshay Sharma, Sara Boyd, Gregory Sallabank

**Affiliations:** 1 Department of Health Behavior and Clinical Sciences, School of Nursing, University of Michigan, 400 N Ingalls St, Ann Arbor, MI, 48109, United States, 1 734-647-0151; 2 Office of Planning and Strategic Initiatives, University of Michigan, Ann Arbor, MI, United States

**Keywords:** South Asian, sexual and gender minorities, men who have sex with men, HIV, risk reduction behavior, pre-exposure prophylaxis, PrEP

## Abstract

**Background:**

South Asian American gay, bisexual, and other men who have sex with men (GBM) experience multiple minority stressors, such as internalized homophobia, racial prejudice, and homonegative discrimination, thereby elevating their risk of HIV. Due to the scarcity of HIV scientific literature focusing on South Asian American GBM, little is known about their use of pre-exposure prophylaxis (PrEP) or the upstream factors influencing its uptake.

**Objective:**

This study seeks to address this gap by describing PrEP use history among South Asian American GBM and comparing PrEP users and nonusers with respect to their demographic and behavioral characteristics, as well as PrEP knowledge, attitudes, stigma, social norms, and self-efficacy—elements that reflect key constructs of the information-motivation-behavioral (IMB) skills model.

**Methods:**

Sexually active South Asian American GBM were recruited from across the United States via social media advertising and peer referral and were administered a Qualtrics survey, for which they received a US $25 Amazon e-gift card. In addition to demographic and behavioral variables, the survey included previously validated scales to measure IMB model constructs. Fisher exact tests and Mann-Whitney *U* tests were conducted to compare the demographic and behavioral characteristics of PrEP users with nonusers, as well as their scores on each scale, respectively.

**Results:**

Of the 103 participants, 34 (33%) were currently using PrEP, 52 (50%) had never used it, and 17 (17%) had discontinued its use. Greater proportions of PrEP users were in open relationships (*P*<.001), had at least 2 male sex partners in the past 6 months (*P*<.001), and had condomless anal sex with at least 2 male partners in the past 6 months (*P*<.001). Frequently cited reasons for never using or discontinuing PrEP included being in or starting a relationship, respectively, and concern about its long-term safety. Consistent with the IMB model, PrEP users exhibited greater PrEP knowledge (*P*<.001), stronger motivational elements (PrEP attitudes *P*=.002; PrEP stigma *P*=.09; PrEP subjective norms *P*=.003; PrEP descriptive norms *P*=.06), and higher PrEP self-efficacy (*P*=.003).

**Conclusions:**

Suboptimal PrEP use among South Asian American GBM, despite high PrEP knowledge, positive attitudes, low stigma, positive social norms, and high PrEP self-efficacy, represents a disconnect between individual readiness and actual use. Future research exploring their sexual health needs through an intersectional lens could help identify possible points for intervention.

## Introduction

### Background

In its fifth decade, HIV continues to disproportionately impact gay, bisexual, and other men who have sex with men (GBM) in the United States, particularly those belonging to racial and ethnic minority subgroups [1,2]. The approval of pre-exposure prophylaxis (PrEP) to prevent HIV, the development of clinical guidelines for those at high risk, and the strategic investments in scaling up PrEP represent critical milestones in slowing the HIV epidemic. Currently, 2 oral PrEP formulations (Truvada and Descovy) are approved by the Food and Drug Administration for daily use among GBM. If used as recommended, daily oral PrEP can reduce the risk of acquiring HIV from sex by up to 99% [3] and from injection drug use by at least 74% [4]. Although PrEP use among GBM has been increasing [5], individual, social, and structural barriers have impeded its widespread adoption. These include a lack of information about PrEP’s purpose and effectiveness, concerns about potential side effects and long-term safety, low HIV risk perception, stigma, and limited access to health care [6-8].

To address the heavy burden of HIV among Black or African American and Hispanic or Latino GBM in the United States [2], concerted efforts have been made by public health agencies to engage them in prevention and care services [9-11]. Several community-informed interventions have also been developed to improve the uptake of PrEP [12-14]. One subgroup that has consistently been neglected in HIV prevention programming and research is South Asian American GBM, individuals who trace their heritage to countries such as India, Pakistan, and Sri Lanka. South Asian Americans are ethnically, linguistically, and culturally diverse and represent a rapidly expanding segment of the overall population [15]. The majority of South Asian Americans are immigrants, with foreign-born individuals comprising 66%, 62%, and 75% of the total Indian, Pakistani, and Sri Lankan populations, respectively, in 2023 [16].

Research from Australia [17,18], Canada [19,20], and the United Kingdom [21,22] has found that South Asian GBM experience multiple minority stressors stemming from their intersectional identities, such as internalized homophobia, racial prejudice, and homonegative discrimination. These stressors have been associated with increased participation in sexual risk behaviors among Black or African American and Hispanic or Latino GBM in the United States [23-25] and may similarly affect South Asian American GBM. Another stressor that could heighten this subgroup’s vulnerability to HIV is immigration [26]. Structural stigma toward sexual minorities and foreign-born individuals has been linked to insufficient HIV prevention knowledge, limited engagement in protective behaviors, and inadequate receipt of health care services among GBM [27,28]. Collectively, these studies underscore the importance of engaging South Asian American GBM in HIV prevention efforts.

Information-motivation-behavioral (IMB) skills model, by Fisher and Fisher, provides a useful framework for conceptualizing and promoting HIV preventive behaviors [29]. Over the years, it has been used to develop interventions promoting consistent condom use [30], to guide content creation for smartphone apps [31], and to evaluate PrEP adherence among racial and ethnic minority GBM [32]. In the context of PrEP use, information refers to the knowledge and understanding an individual has about this HIV prevention tool, including its safety, efficacy, and potential contraindications. Motivation involves an individual’s desire and willingness to use PrEP, shaped by elements such as attitudes, stigma, and social norms. The latter are often categorized into subjective norms (ie, perceived expectations and opinions of others regarding a behavior) and descriptive norms (ie, actual behavior of others). Behavioral skills encompass an individual’s self-efficacy to use PrEP as recommended (ie, belief in their ability to perform the behavior successfully). These constructs are interconnected and reciprocally interact to determine whether an individual will initiate and sustain PrEP use [33].

### Objective

Due to the scarcity of HIV scientific literature focusing on South Asian American GBM, little is known about their use of PrEP or the upstream factors influencing its uptake. This study seeks to address this gap by describing PrEP use history among South Asian American GBM and comparing PrEP users and nonusers with respect to their demographic and behavioral characteristics, as well as PrEP knowledge, attitudes, stigma, social norms, and self-efficacy, elements that reflect key constructs of the IMB model [33]. Our results could help lay a strong foundation for designing and evaluating novel, culturally relevant PrEP-related interventions for South Asian American GBM at elevated risk of HIV.

## Methods

### Study Design

Data for these analyses were derived from “Ujala,” a pilot web-based survey study conducted from April to July 2022 among South Asian American GBM residing in different regions of the country. The study’s name, which means “light,” “radiant,” or “bright” in multiple South Asian languages, was chosen to pique curiosity and bolster engagement among potential participants. “Ujala” was designed as a preliminary investigation of HIV-related risk and preventive behaviors among South Asian American GBM. Pilot studies are well suited for exploratory research to identify recruitment avenues, estimate parameters, and inform subsequent larger-scale studies, with recommended sample sizes ranging from 50 to 150 participants [34-36]. Accordingly, we aimed to recruit approximately 100 participants, corresponding to the midpoint of the recommended range for pilot studies.

### Participant Recruitment

Participants were recruited by requesting administrators of web-based South Asian American LGBTQ+ (lesbian, gay, bisexual, transgender/transsexual, queer, and other minority sexual orientations and gender identities) groups (eg, MASALA Boston, Trikone Bay Area, and Khush ATX) to post study advertisements on their social media channels. Individuals who clicked through to the study’s landing page, programmed in Qualtrics, were requested to provide electronic informed consent and were screened for eligibility. Only those who identified as male, were at least 18 years of age, resided in the United States, were of legal age to provide consent in their state or territory of residence, identified as South Asian, had at least 1 male sex partner in the past 6 months, had never been diagnosed with HIV, and were willing to provide their name and email address to receive a US $25 Amazon e-gift card were eligible to participate. Eligible individuals were directed to a Qualtrics survey that collected information on demographic and behavioral characteristics as well as on several PrEP-related variables (Multimedia Appendix 1). The final page of the survey included a link to the study’s landing page that could be shared with potential participants in their networks.

### Survey Measures

#### Demographic Characteristics

Demographic data collected from participants included their age, education level, employment status, health insurance coverage, state or territory of residence in the United States, nativity status, duration of residence in the United States, sexual orientation, and relationship type. Those in a relationship were asked whether they had formulated a sexual agreement, that is, a mutual understanding regarding sex with outside partners, and, if so, whether they were in an open relationship, wherein sex with outside partners was permitted by mutual agreement, or in a closed relationship, wherein sex with outside partners was not permitted by mutual agreement.

#### Behavioral Characteristics

Behavioral data collected from participants included their number of male sex partners in the past 6 months, number of male partners with whom they had condomless anal sex in the past 6 months, HIV serostatus of the partners with whom they had anal sex in the past 6 months, alcohol or drug use immediately before or during sex in the past 6 months, and HIV testing history. Condomless anal sex was described as insertive or receptive anal sex during which a condom was not used the entire time.

#### PrEP Use History

Participants were asked whether they had ever used PrEP to reduce their risk of acquiring HIV, and, if so, whether they were currently using PrEP. Those who had never used PrEP or had discontinued its use were asked to indicate their reasons from prespecified options. They were also presented with open-ended text fields in which they could enter other reasons.

#### Information: PrEP Knowledge

Knowledge was assessed using a 13-item scale (Cronbach *α*=0.95) that asked participants to distinguish between correct and incorrect information about PrEP [33]. It included statements such as “PrEP can be taken by people who already have HIV.” Response options for this scale were as follows: “True,” “False,” and “Don’t know.” Items answered correctly were assigned a value of 1, and those answered incorrectly or marked as “Don’t know” were assigned a value of 0.

#### Motivation: PrEP Attitudes, Stigma, and Social Norms

Attitudes were assessed using a 5-item scale (Cronbach *α*=0.83) that included statements such as “People who take PrEP are responsible” [33]. Stigma was assessed using a 5-item scale (Cronbach *α*=0.65) that included statements such as “People who take PrEP are promiscuous” [33]. Subjective norms were assessed using a 6-item scale (Cronbach *α*=0.93) that included statements such as “My friends would think it was responsible if I used PrEP” [33]. Descriptive norms were assessed using a 6-item scale (Cronbach *α*=0.89) that included statements such as “My friends would consider taking PrEP” [33]. Response options for each scale were as follows: “Strongly disagree” (assigned a value of 1), “Disagree” (assigned a value of 2), “Neutral” (assigned a value of 3), “Agree” (assigned a value of 4), and “Strongly agree” (assigned a value of 5).

#### Behavioral Skills: PrEP Self-Efficacy

Self-efficacy was assessed using an 8-item scale (Cronbach *α*=0.88) that asked participants about how challenging or straightforward it would be to engage in behaviors related to PrEP use [33]. It included statements such as “Visit a doctor every 3 months for routine screenings.” Response options for this scale were as follows: “Very hard to do” (assigned a value of 1), “Somewhat hard to do” (assigned a value of 2), “Somewhat easy to do” (assigned a value of 3), and “Very easy to do” (assigned a value of 4).

### Analytic Sample

Specifics regarding participant recruitment, including the number of individuals at each stage (ie, click-throughs to the study’s landing page, provision of informed consent, and eligibility screening), have been described elsewhere [37]. Of the 140 individuals who began the survey, 103 (74%) provided complete data on all PrEP-related variables of interest and were included in the analytic sample. Missing data were handled using complete-case analysis, ensuring that all reported results are based solely on observed participant responses. No differences in demographic or behavioral characteristics were observed between individuals included in the analytic sample and those excluded.

### Data Analysis

Statistical analyses were conducted using SAS. Descriptive statistics were calculated to summarize the demographic and behavioral characteristics of the participants, overall and stratified by current PrEP use. Fisher exact tests, which are useful when dealing with small sample sizes, were conducted to compare PrEP users and nonusers with respect to each characteristic. Reasons indicated by the participants for never using or discontinuing PrEP were also summarized.

Responses to items on the PrEP knowledge, attitudes, stigma, subjective norms, descriptive norms, and self-efficacy scales were summed to generate a score for each scale for each participant. Medians and IQRs were calculated for each score to characterize their distribution, overall and stratified by current PrEP use. Mann-Whitney *U* tests were conducted to compare PrEP users and nonusers with respect to each scale score.

### Ethical Considerations

Study materials and procedures were reviewed and approved by the institutional review board at the University of Michigan (HUM00209310). Electronic informed consent was obtained from all individual participants included in the study. Survey responses were deidentified, as names and email addresses used for incentive distribution were collected through a separate Qualtrics form and were not linked to survey responses. Participant data were stored and analyzed on secure, password-protected platforms at the University of Michigan approved for sensitive human-subjects research data, with access restricted to institutional review board–approved study team members. Participants who completed at least 80% of the survey were thanked for their contribution via email and sent a US $25 Amazon e-gift card.

## Results

### Demographic and Behavioral Characteristics

Table 1 summarizes the demographic and behavioral characteristics of the 103 participants, overall and stratified by current PrEP use. Regarding ethnic origin, the majority were Indian (94, 91%), followed by Pakistani (7, 7%), Sri Lankan (1, 1%), and Sindhi (1, 1%). Ages ranged from 22 to 57 years, with a median of 34 (IQR 31-42) years. The majority held a master’s or doctoral degree, were employed full time or part time, were born outside the United States, had been residing in the United States for more than 10 years, and identified as gay. Of those who were partnered, almost half were in an open relationship, and almost one-quarter were in a relationship wherein no sexual agreement had been formulated. In the past 6 months, almost two-thirds had at least 2 male sex partners, and almost one-third had condomless anal sex with at least 2 male partners. Additionally, almost one-third had not been tested for HIV in the past year.

**Table 1.** Demographic and behavioral characteristics of 103 South Asian American gay, bisexual, and other men who have sex with men (GBM).

Characteristic	Overall sample (n=103), n (%)	Currently using PrEP^a^ (n=34), n (%)	Not currently using PrEP (n=69), n (%)	*P* value^b^
Age (y)				.33
18‐29	23 (22)	9 (26)	14 (20)	
30‐39	49 (48)	18 (53)	31 (45)	
≥40	31 (30)	7 (21)	24 (35)	
Education level				.62
High school or bachelor’s degree	23 (22)	9 (26)	14 (20)	
Master’s or doctoral degree	80 (78)	25 (74)	55 (80)	
Employment status				.66
Employed (full time or part time)	87 (84)	28 (82)	59 (86)	
Student (full time or part time)	11 (11)	5 (15)	6 (9)	
Not employed	5 (5)	1 (3)	4 (6)	
Health insurance				.53
Private health plan	91 (88)	29 (85)	62 (90)	
Other^c^	12 (12)	5 (15)	7 (10)	
Region of residence				.05
Northeast	17 (17)	6 (18)	11 (16)	
Midwest	31 (30)	16 (47)	15 (22)	
South	38 (37)	9 (26)	29 (42)	
West	17 (17)	3 (9)	14 (20)	
Nativity status				.33
Born in the United States	24 (23)	10 (29)	14 (20)	
Born outside the United States	79 (77)	24 (71)	55 (80)	
Duration of residence in the United States				.81
Since birth	16 (16)	6 (18)	10 (14)	
>10 years	50 (49)	14 (41)	36 (52)	
6‐10 years	19 (18)	7 (21)	12 (17)	
1‐5 years	14 (14)	6 (18)	8 (12)	
<1 year	4 (4)	1 (3)	3 (4)	
Sexual orientation				.43
Gay	92 (89)	30 (88)	62 (90)	
Bisexual	8 (8)	2 (6)	6 (9)	
Other^d^	3 (3)	2 (6)	1 (1)	
Relationship type				<.001
Single	54 (52)	19 (56)	35 (51)	
Partnered, open relationship	22 (21)	14 (41)	8 (12)	
Partnered, closed relationship	16 (16)	0 (0)	16 (23)	
Partnered, no sexual agreement	11 (11)	1 (3)	10 (14)	
Number of male sex partners in the past 6 months				<.001
1	39 (38)	2 (6)	37 (54)	
≥2	64 (62)	32 (94)	32 (46)	
Had condomless anal sex with at least 2 male partners in the past 6 months				<.001
Yes	30 (29)	23 (68)	7 (10)	
No^e^	73 (71)	11 (32)	62 (90)	
Had anal sex with a male partner living with HIV in the past 6 months				.09
Yes	6 (6)	4 (12)	2 (3)	
No^f^	97 (94)	30 (88)	67 (97)	
Used alcohol or drugs immediately before or during sex in the past 6 months				.10
Yes	28 (27)	13 (38)	15 (22)	
No	75 (73)	21 (62)	54 (78)	
Tested for HIV in the past year				<.001
Yes	73 (71)	34 (100)	39 (57)	
No^g^	30 (29)	0 (0)	30 (43)	

a PrEP: pre-exposure prophylaxis.

b Two-sided *P* value from Fisher exact test.

c Includes 5 insured under the Affordable Care Act, 4 insured under Medicare or Medicaid, 1 insured under the Veterans Affairs health plan, and 2 insured under some other health plan.

d Includes 2 queer and 1 pansexual.

e Includes 31 individuals who had condomless anal sex with 1 male partner and 42 who did not have condomless anal sex.

f Includes 66 individuals who had anal sex with a male partner without HIV, 8 who had anal sex with a male partner of unknown HIV serostatus, and 23 who did not have anal sex.

g Includes 24 who tested for HIV more than 1 year ago and 6 who never tested for HIV.

### PrEP Use History

Regarding PrEP use among the 103 participants, 34 (33%) were currently using it, 52 (50%) had never used it, and 17 (17%) had discontinued its use. Greater proportions of PrEP users were in open relationships, had at least 2 male sex partners in the past 6 months, and had condomless anal sex with at least 2 male partners in the past 6 months compared to nonusers. The most frequently cited reasons for never using PrEP included being in a relationship (17/52, 33%), low HIV risk perception (15/52, 29%), and concern about its long-term safety (7/52, 13%). The most frequently cited reasons for discontinuing PrEP included starting a relationship (7/17, 41%), limited coverage under one’s health insurance plan (3/17, 18%), and concern about its long-term safety (2/17, 12%). Subgroup analyses among 63 participants who likely met clinical indications for PrEP (ie, had condomless anal sex in the past 6 months or had anal sex with a partner living with HIV in the past 6 months) revealed that 29 (46%) were currently using it, 22 (35%) had never used it, and 12 (19%) had discontinued its use.

### PrEP Knowledge, Attitudes, Stigma, Social Norms, and Self-Efficacy

**Figure 1.**
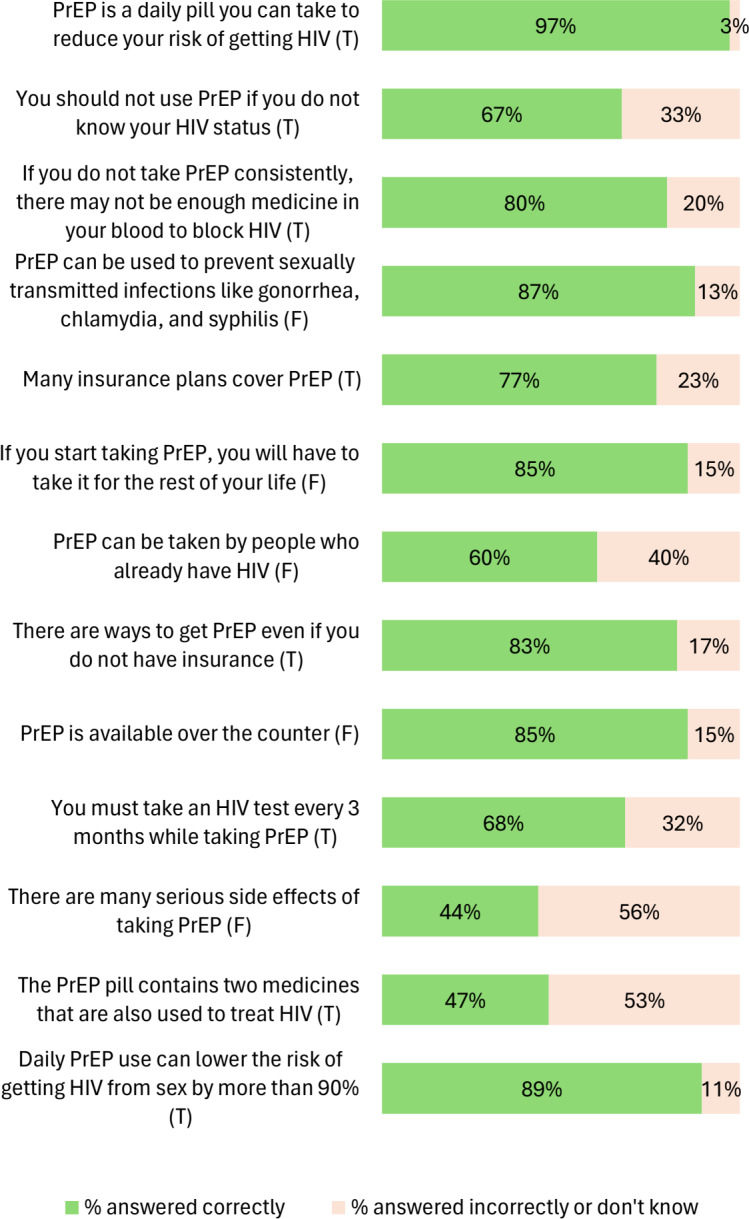
Responses to items on the pre-exposure prophylaxis (PrEP) knowledge scale among 103 South Asian American gay, bisexual, and other men who have sex with men (GBM).

Figure 1 depicts the proportions of each item on the PrEP knowledge scale that were answered correctly and those answered incorrectly or marked as “Don’t know” by the 103 participants. The majority answered each question correctly, except for the ones asking if PrEP has serious side effects and if the PrEP pill contains a combination of medications also used to treat HIV.

**Figure 2.**
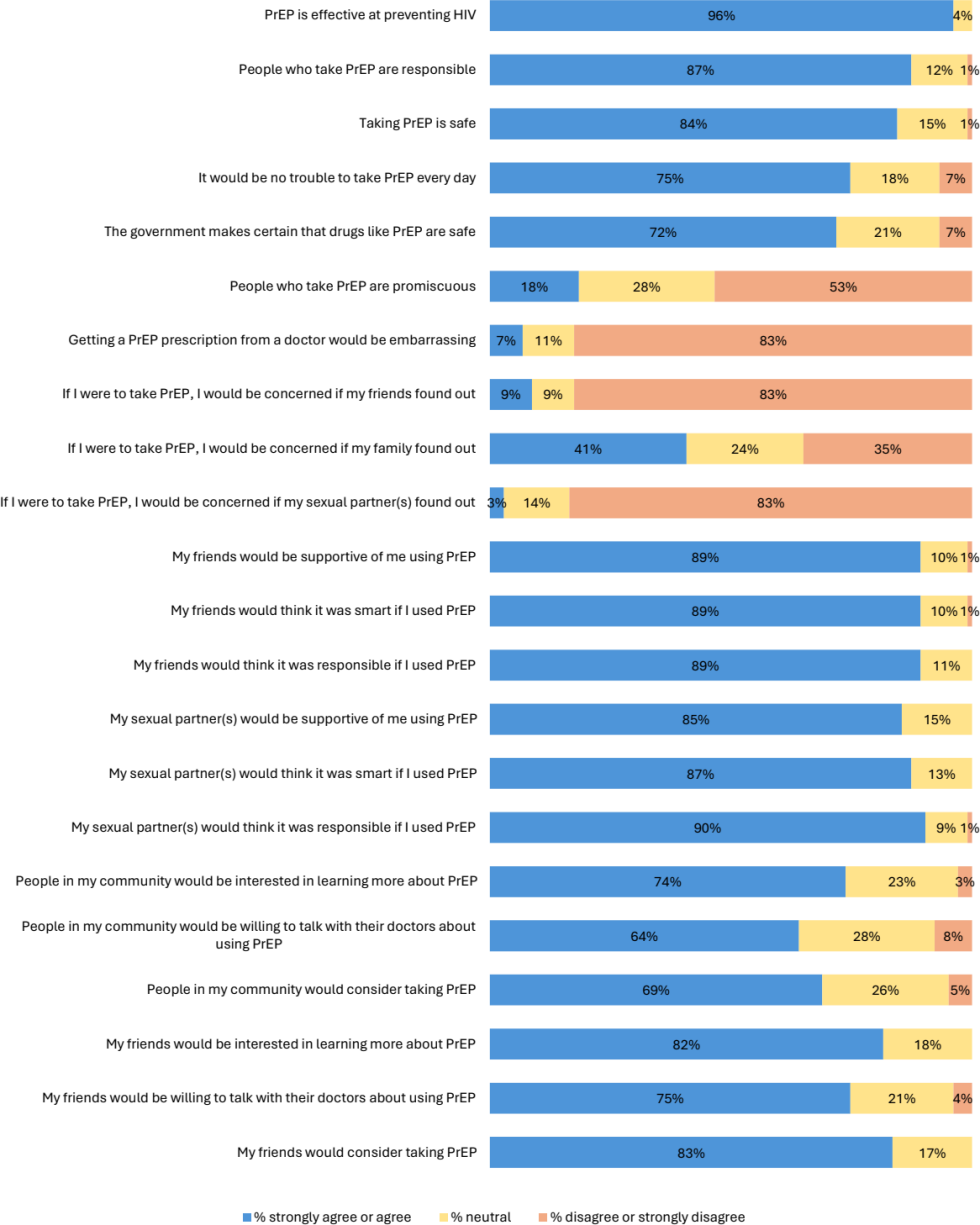
Responses to items on the pre-exposure prophylaxis (PrEP) attitudes, stigma, subjective norms, and descriptive norms scales among 103 South Asian American gay, bisexual, and other men who have sex with men (GBM).

Figure 2 summarizes the proportions of each item on the PrEP attitudes, stigma, subjective norms, and descriptive norms scales that were strongly agreed or agreed with, marked as neutral, and disagreed or strongly disagreed with by the 103 participants. The majority strongly agreed or agreed with each item on the PrEP attitudes scale, disagreed or strongly disagreed with each item on the PrEP stigma scale (except for the one about disclosure of PrEP use to family members), and strongly agreed or agreed with each item on the subjective and descriptive norms scales.

**Figure 3.**
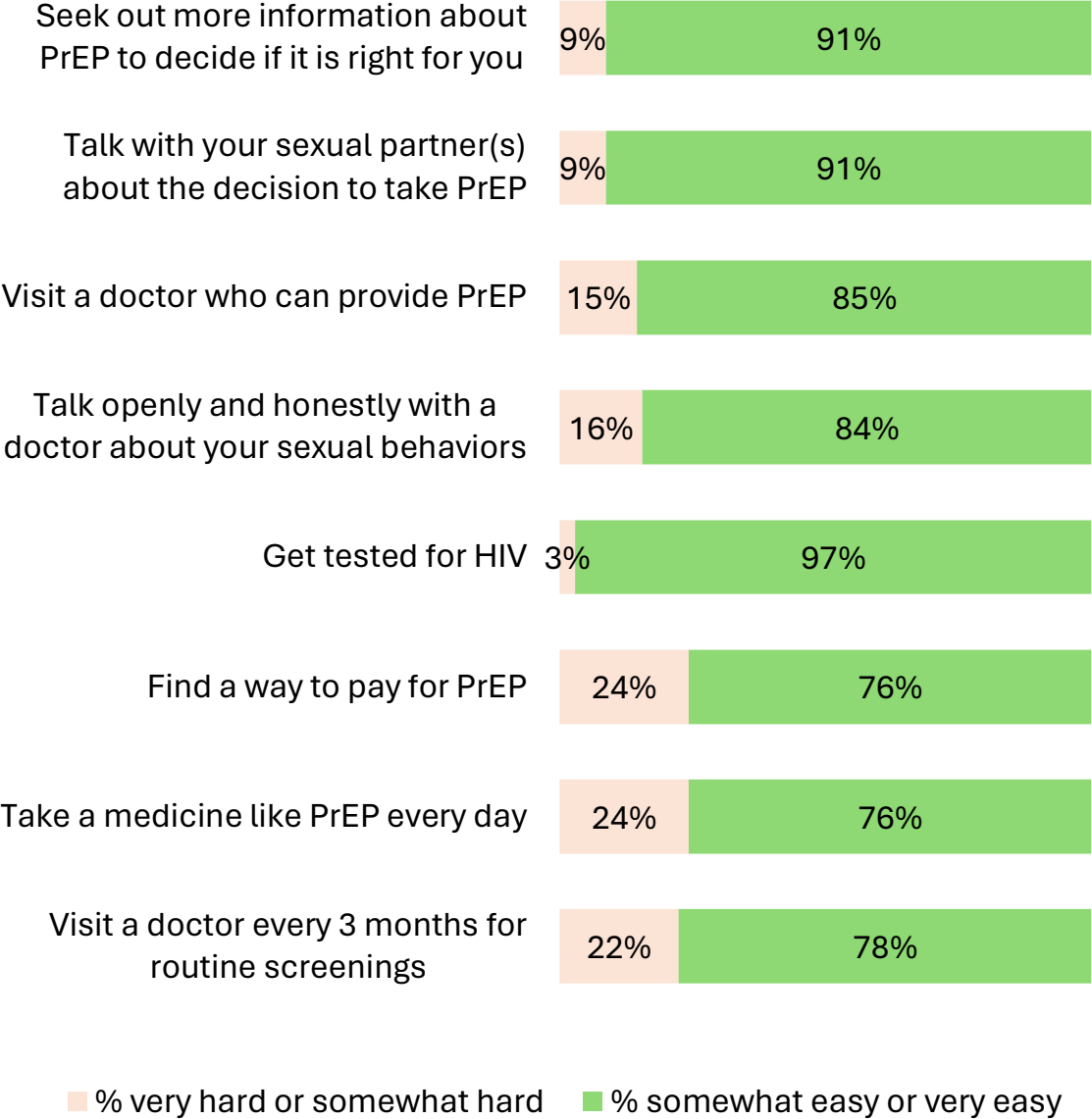
Responses to items on the pre-exposure prophylaxis (PrEP) self-efficacy scale among 103 South Asian American gay, bisexual, and other men who have sex with men (GBM).

Figure 3 depicts the proportions of each item on the PrEP self-efficacy scale that were deemed very hard or somewhat hard to perform and somewhat easy or very easy to perform by the 103 participants. The majority indicated that each task was somewhat easy or very easy to perform.

Table 2 summarizes the distributions of scores on the PrEP knowledge, attitudes, stigma, subjective norms, descriptive norms, and self-efficacy scales completed by the 103 participants, overall and stratified by current PrEP use. The distribution of scores on the PrEP knowledge scale was skewed to the left, indicating generally high knowledge. Greater knowledge was noted among PrEP users versus nonusers. The distribution of scores on the PrEP attitudes scale was skewed to the left, indicating generally positive attitudes. More positive attitudes were noted among PrEP users versus nonusers. The distribution of scores on the PrEP stigma scale was skewed to the right, indicating generally low stigma. No differences in stigma were noted between PrEP users and nonusers. The distribution of scores on the PrEP subjective norms scale was skewed to the left, indicating generally positive subjective norms. More positive subjective norms were noted among PrEP users versus nonusers. The distribution of scores on the PrEP descriptive norms scale was skewed to the left, indicating generally positive descriptive norms. No differences in descriptive norms were noted between PrEP users and nonusers. The distribution of scores on the PrEP self-efficacy scale was skewed to the left, indicating generally high self-efficacy. Greater self-efficacy was noted among PrEP users versus nonusers.

**Table 2.** Pre-exposure prophylaxis (PrEP) knowledge, attitudes, stigma, subjective norms, descriptive norms, and self-efficacy among 103 South Asian American gay, bisexual, and other men who have sex with men (GBM).

Scale	Overall sample (n=103), median (IQR)	Currently using PrEP (n=34), median (IQR)	Not currently using PrEP (n=69), median (IQR)	Mann-Whitney *U* test results		
				*z* score	*r* value	*P* value^a^
PrEP knowledge^b^	11 (8‐12)	12 (11‐13)	9 (7‐11)	5.30	.52	<.001
PrEP attitudes^c^	21 (19‐24)	23 (21‐25)	20 (19‐24)	3.11	.31	.002
PrEP stigma^d^	11 (9‐13)	10 (9‐12)	11 (9‐14)	1.72	.17	.09
PrEP subjective norms^e^	27 (24‐30)	30 (27‐30)	24 (24‐30)	3.02	.30	.003
PrEP descriptive norms^f^	24 (22‐27)	24 (24‐27)	24 (20‐26)	1.87	.19	.06
PrEP self-efficacy^g^	27 (23‐31)	29 (27‐32)	25 (22‐30)	3.02	.30	.003

a Two-sided *P *value from Mann-Whitney *U* test.

b Assessed using a 13-item scale potentially ranging from 0 to 13, with higher scores indicating greater knowledge.

c Assessed using a 5-item scale potentially ranging from 5 to 25, with higher scores indicating more positive attitudes.

d Assessed using a 5-item scale potentially ranging from 5 to 25, with higher scores indicating greater stigma.

e Assessed using a 6-item scale potentially ranging from 6 to 30, with higher scores indicating more positive subjective norms.

f Assessed using a 6-item scale potentially ranging from 6 to 30, with higher scores indicating more positive descriptive norms.

g Assessed using an 8-item scale potentially ranging from 8 to 32, with higher scores indicating greater self-efficacy.

## Discussion

### Principal Findings

Our study addresses an important gap in the HIV scientific literature by examining PrEP utilization among South Asian American GBM and the potential role of theory-based behavioral antecedents, specifically information, motivation, and behavioral skills, in shaping its uptake. Consistent with the IMB model, greater knowledge, more positive attitudes and subjective norms, and greater self-efficacy were noted among PrEP users compared to nonusers, paralleling studies with other racial and ethnic minority GBM in the United States [38-42], and providing empirical support for the IMB model in explaining PrEP use behavior in this subgroup. Our results indicate suboptimal PrEP use, with only one-third of all participants and less than half of those who likely met clinical indications for PrEP reporting use at the time of the survey. Notably, the underuse of PrEP in our sample occurred despite high PrEP knowledge, positive attitudes, low stigma, positive social norms, and high PrEP self-efficacy, suggesting a disconnect between individual readiness and uptake. Theoretically, these findings suggest that within the IMB framework, favorable levels of information, motivation, and behavioral skills may not fully translate into PrEP uptake in the absence of enabling contextual factors. Our study underscores the need for targeted strategies that address personal, cultural, and structural barriers that may be limiting access to, initiation of, and sustained use of PrEP among South Asian American GBM.

Although PrEP uptake among GBM in the United States has been on the rise, racial and ethnic disparities persist. In 2023, 38% of 1125 non-Hispanic Black or African American GBM and 43% of 1477 Hispanic or Latino GBM participating in the National HIV Behavioral Surveillance were using PrEP, compared to 51% of 1709 non-Hispanic White GBM [43]. Interestingly, 60% of 187 non-Hispanic Asian GBM were using PrEP, higher than any other racial or ethnic category [43]. Disaggregated data for South Asian American GBM were not reported, a disconcerting and recurring issue in the HIV surveillance literature, as it precludes our understanding of their unmet HIV prevention needs. Our current PrEP use estimates of 33% among all participants and 46% among those who likely met clinical indications for PrEP offer preliminary evidence to fill this gap. They also shed light on the progress made thus far and the need to promote equitable PrEP implementation to ensure that South Asian American GBM are not underserved in HIV prevention efforts.

Regarding variations in characteristics between PrEP users and nonusers, no differences were observed across any demographic characteristic, except for relationship type. Although similar proportions of GBM in each category were single, more PrEP users were in open relationships, and more nonusers were in closed relationships or did not have mutually formulated sexual agreements. This suggests that PrEP uptake may be influenced by relationship dynamics and communication, with GBM in open relationships potentially engaging in explicit conversations about sexual boundaries and HIV risk with their partners [44,45]. South Asian American GBM in relationships that are not strictly monogamous or include periods of nonmonogamy, whether consensual or not, remain susceptible to HIV and warrant attention in HIV prevention research and programming.

Exploring the reasons indicated by our participants for never using PrEP and discontinuing its use, the most frequently reported were being in a relationship and starting a relationship, respectively. Racial and ethnic minority GBM in the United States frequently cite these as normative justifications for avoiding or stopping PrEP [46,47]. Modeling suggests that a significant proportion of new HIV infections among partnered GBM occur within relationships [48,49], often due to reduced risk perception stemming from assumptions of exclusivity, leading to decreased condom use [50,51] and less frequent testing [52,53]. Decisions regarding PrEP use among partnered GBM should ideally be based on actual risk behaviors, open and honest communication, and clarity around sexual agreements. Our findings have practical implications for PrEP programming within couple-based and relationship-focused interventions. Empowering partnered South Asian American GBM to make informed, realistic, and mutually agreed-upon decisions about using PrEP, for example, through couples counseling, peer-led discussions, and digital resources, is important for safeguarding their sexual health.

Another frequently cited reason for never using or discontinuing PrEP was a concern about its long-term safety, which also parallels findings from other studies with GBM [54,55]. Although both FDA-approved oral formulations for PrEP are generally regarded to be safe and well tolerated [56], especially with regular monitoring, the possibility of long-term side effects from extended use exists. Truvada has been linked to decreased kidney function [57] and small reductions in bone mineral density [58], whereas Descovy has been associated with weight gain and elevated lipid levels [59]. However, these adverse events are rare, as confirmed by multiple clinical trials [60,61]. South Asian Americans have a strong cultural connection to traditional medicinal systems, such as Ayurveda, Siddha, and Unani, and may be reluctant to embrace the long-term use of allopathic drugs [62,63]. Our results highlight the need for clear, culturally informed PrEP communication strategies and suggest that tailored messaging emphasizing the safety of PrEP, possibly comparing it to other common long-term medications such as those for diabetes and hypertension, might help alleviate such concerns.

Shifting focus to the information construct of the IMB model, our sample demonstrated generally high PrEP knowledge, indicating a strong baseline understanding of this HIV prevention tool. Notably, most of our participants were highly educated, with more than three-fourths holding a master’s or doctoral degree. Yet, more than half responded incorrectly or indicated uncertainty on questions about whether PrEP has serious side effects and whether the PrEP pill contains a combination of medications also used to treat HIV, highlighting deficits in specific knowledge areas. Many GBM have reported receiving PrEP information from their peers, who, while accessible, sometimes provide incomplete or inaccurate details [64]. Others have identified their health care providers as initial sources of PrEP information, acknowledging them as trusted authorities who dispel misconceptions while reinforcing its safety and effectiveness [64]. South Asian GBM residing in Western countries are often reluctant to disclose their sexual orientation to medical professionals due to familial, cultural, and social pressures [17,19,21], limiting their opportunities to learn about HIV prevention options, including PrEP. Our findings underscore the importance of strengthening accurate, provider-delivered PrEP education and improving access to affirming health care environments. Future studies could explore how perceived and experienced stigmas influence clinical interactions and identify practical strategies to further enhance PrEP knowledge.

Focusing on the motivation construct of the IMB model, our participants harbored generally positive attitudes, low stigma, and generally positive subjective and descriptive norms regarding PrEP, suggesting favorable perceptions and adequate social support to consider or initiate its use. The only exception was that more than two-fifths were concerned about their families finding out if they were using PrEP. This may reflect fears of being questioned about their sexual orientation, being judged as promiscuous, or being perceived to already have HIV. Family plays a pivotal role in South Asian culture, and for GBM, relationships often exist in a delicate balance between personal identity, cultural expectations, and societal conformity [18,20,22]. Given the potential for conflict between individual autonomy in health-related decisions and familial influences on those decisions, creating supportive spaces for open dialogue could help mitigate concerns about the inadvertent disclosure of PrEP use.

Regarding the behavioral skills construct of the IMB model, our sample exhibited generally high PrEP self-efficacy, indicating a strong sense of confidence in navigating behaviors related to accessing, initiating, and sustaining PrEP use. However, almost a quarter reported that finding a way to pay for PrEP, taking a pill every day, and visiting a health care provider every 3 months for routine screenings would be arduous. Cost, adherence, and ongoing clinic visits are practical barriers, but they are not insurmountable. The first could be addressed by investigating insurance coverage options and public health initiatives that offer PrEP at reduced or no cost [65,66]. The second could be mitigated through considering medication adherence programs that provide support in the form of counseling and reminders [67,68] or by exploring long-acting injectable PrEP formulations (Apretude and Yeztugo) [69]. The third could be addressed by using telehealth delivery models to reduce structural and logistical impediments [70,71].

### Study Limitations

Despite our understanding that “Ujala” is among the few studies focusing on the sexual health of South Asian American GBM, it is not without limitations. Participants were recruited from web-based South Asian American LGBTQ+ groups and via peer referral, thereby excluding individuals with limited engagement in web-based LGBTQ+ communities and social networks. Individuals self-selected into the study and were required to provide their name and email address in a separate Qualtrics form to receive the incentive, resulting in the study not being fully anonymous and potentially introducing selection bias. More than three-fourths of the participants were born outside the United States and had a postgraduate degree, which may limit the generalizability of our findings. However, these characteristics should be interpreted in the context of the broader South Asian American population, which is predominantly composed of immigrants [16] and has been consistently reported to have high levels of educational attainment [72]. Social desirability bias may have resulted in an underreporting of sexual risk behaviors and overreporting of HIV prevention behaviors, but we do not anticipate substantial misreporting, as our participants self-completed the survey and could skip questions that made them uncomfortable. The cross-sectional design of this study precludes us from commenting on temporal associations between the IMB model constructs and PrEP use. Additionally, the scope of the data collected in the study and the sample size limited our ability to conduct additional subgroup analyses. Future studies with larger samples of South Asian American GBM could examine potential variations in PrEP use as well as PrEP knowledge, attitudes, stigma, social norms, and self-efficacy across different subgroups. Finally, the previously validated scales that we used to measure IMB model constructs were developed before the availability of long-acting injectable PrEP formulations and therefore did not capture knowledge, motivation, or self-efficacy related to this option. Nonetheless, they provided reliable information on these constructs with respect to daily oral PrEP in our sample.

### Conclusions

Our study makes a noteworthy contribution by describing PrEP use among sexually active South Asian American GBM, exploring differences in demographic and behavioral characteristics between PrEP users and nonusers, and illuminating reasons for avoiding or stopping PrEP, issues that have not been well characterized in the HIV scientific literature. It also highlights the use of the IMB model in explaining PrEP use behavior in this historically understudied subgroup and underscores the importance of addressing informational, motivational, and behavioral skills-related barriers to improve PrEP uptake. As a pilot study with a relatively small, convenience-based sample, these findings provide preliminary evidence and should be interpreted as an initial exploration. Nonetheless, they offer a foundation for future larger-scale research examining PrEP use and its behavioral determinants among South Asian American GBM across diverse settings in the United States. Subsequent work to better understand the underlying causes of suboptimal PrEP use in this subgroup conducted through an intersectional lens could help identify possible points for intervention.

## Supplementary material

10.2196/88920 Multimedia Appendix 1 Ujala study instruments.

## Abbreviations

GBM: gay, bisexual, and other men who have sex with men
IMB: information-motivation-behavioral skills
LGBTQ+: lesbian, gay, bisexual, transgender/transsexual, queer, and other minority sexual orientations and gender identities
PrEP: pre-exposure prophylaxis

## References

[1] (2024). HIV surveillance supplemental report: estimated HIV incidence and prevalence in the United States, 2018–2022. https://stacks.cdc.gov/view/cdc/156513.

[2] (2024). HIV surveillance report: diagnoses, deaths, and prevalence of HIV in the United States and 6 territories and freely associated states, 2022. https://stacks.cdc.gov/view/cdc/156509.

[3] Anderson PL, Glidden DV, Liu A (2012). Emtricitabine-tenofovir concentrations and pre-exposure prophylaxis efficacy in men who have sex with men. Sci Transl Med.

[4] Choopanya K, Martin M, Suntharasamai P (2013). Antiretroviral prophylaxis for HIV infection in injecting drug users in Bangkok, Thailand (the Bangkok Tenofovir Study): a randomised, double-blind, placebo-controlled phase 3 trial. The Lancet.

[5] Sullivan PS, Juhasz M, DuBose SN (2025). Association of state-level PrEP coverage and new HIV diagnoses in the USA from 2012 to 2022: an ecological analysis of the population impact of PrEP. Lancet HIV.

[6] O’Neil AM, Hubach RD, Owens C, Walsh JL, Quinn KG, John SA (2025). Determinants of HIV pre-exposure prophylaxis (PrEP) use among men who have sex with men (MSM) living in rural areas of the United States: a scoping review framed by the PrEP care continuum. J Rural Health.

[7] Matos LA, Janek SE, Holt L, Ledbetter L, Gonzalez-Guarda RM (2024). Barriers and facilitators along the PrEP continuum of care among Latinx sexual minoritized men and transgender women: a systematic review. AIDS Behav.

[8] Russ S, Zhang C, Liu Y (2021). Pre-exposure prophylaxis care continuum, barriers, and facilitators among Black men who have sex with men in the United States: a systematic review and meta-analysis. AIDS Behav.

[9] Self KJ, Johnson A, Craker L (2025). Strengthening PrEP services at community-based organizations for Latinx men who have sex with men: an implementation science approach. Arch Public Health.

[10] Beyrer C, Adimora AA, Hodder SL (2021). Call to action: how can the US Ending the HIV Epidemic initiative succeed?. The Lancet.

[11] Ramos SR, Nelson LE, Jones SG, Ni Z, Turpin RE, Portillo CJ (2021). A state of the science on HIV prevention over 40 years among Black and Hispanic/Latinx communities. J Assoc Nurses AIDS Care.

[12] Escarfuller SG, Mitchell JW, Sanchez M (2024). HIV prevention intervention-related research with adult, sexual minority Hispanic men in the United States: a systematic review. J Racial Ethn Health Disparities.

[13] Turpin RE, Hawthorne DJ, Rosario AD (2022). Pre-exposure prophylaxis interventions among Black sexual minority men: a systematic literature review. Int J Environ Res Public Health.

[14] Wang Y, Mitchell JW, Zhang C, Liu Y (2022). Evidence and implication of interventions across various socioecological levels to address pre-exposure prophylaxis uptake and adherence among men who have sex with men in the United States: a systematic review. AIDS Res Ther.

[15] Rico B, Hahn JK, Spence C (2023). Asian Indian was the largest Asian alone population group in 2020. US Census Bureau.

[16] Krogstad JM, Im C Key facts about Asians in the US 2025. Pew Research Center.

[17] Phillips TR, Medland N, Chow EPF (2020). “Moving from one environment to another, it doesn’t automatically change everything”. Exploring the transnational experience of Asian-born gay and bisexual men who have sex with men newly arrived in Australia. PLOS One.

[18] Smith G (2012). Sexuality, space and migration: South Asian gay men in Australia. N Z Geog.

[19] Hart TA, Sharvendiran R, Chikermane V, Kidwai A, Grace D (2023). At the intersection of homophobia and racism: sociocultural context and the sexual health of South Asian Canadian gay and bisexual men. Stigma Health.

[20] Rana M, Nath R, Saewyc E (2024). Parental accepting and rejecting behaviors: experiences of South Asian gay and bisexual men in Canada. Fam Relat.

[21] Jaspal R, Lopes B, Jamal Z, Yap C, Paccoud I, Sekhon P (2019). HIV knowledge, sexual health and sexual behaviour among Black and minority ethnic men who have sex with men in the UK: a cross-sectional study. Sex Health.

[22] Mitha K, Ali S, Koc Y (2021). Challenges to identity integration amongst sexual minority British Muslim South Asian men. J Community Appl Soc Psychol.

[23] Diaz JE, Schrimshaw EW, Tieu HV, Nandi V, Koblin BA, Frye V (2020). Acculturation as a moderator of HIV risk behavior correlates among Latino men who have sex with men. Arch Sex Behav.

[24] Han C, Ayala G, Paul JP, Boylan R, Gregorich SE, Choi KH (2015). Stress and coping with racism and their role in sexual risk for HIV among African American, Asian/Pacific Islander, and Latino men who have sex with men. Arch Sex Behav.

[25] Amola O, Grimmett MA (2015). Sexual identity, mental health, HIV risk behaviors, and internalized homophobia among Black men who have sex with men. J Couns Dev.

[26] Santoso D, Asfia SKBM, Mello MB (2022). HIV prevalence ratio of international migrants compared to their native-born counterparts: a systematic review and meta-analysis. EClinicalMedicine.

[27] Ross J, Cunningham CO, Hanna DB (2018). HIV outcomes among migrants from low-income and middle-income countries living in high-income countries: a review of recent evidence. Curr Opin Infect Dis.

[28] Pachankis JE, Hatzenbuehler ML, Berg RC (2017). Anti-LGBT and anti-immigrant structural stigma: an intersectional analysis of sexual minority men’s HIV risk when migrating to or within Europe. J Acquir Immune Defic Syndr.

[29] Fisher JD, Fisher WA, Shuper PA, DiClemente RJ, Crosby RA, Kegler MC (2009). Emerging Theories in Health Promotion Practice and Research.

[30] Chang SJ, Choi S, Kim SA, Song M (2014). Intervention strategies based on Information-Motivation-Behavioral Skills Model for health behavior change: a systematic review. Asian Nurs Res.

[31] Aliabadi N, Carballo-Dieguez A, Bakken S (2015). Using the information-motivation-behavioral skills model to guide the development of an HIV prevention smartphone application for high-risk MSM. AIDS Educ Prev.

[32] Knox J, Kutner BA, Shiau S (2022). Assessing the information-motivation-behavioral skills model to predict pre-exposure prophylaxis adherence among Black men who have sex with men and transgender women in a community setting in New York city. AIDS Behav.

[33] Walsh JL (2019). Applying the information-motivation-behavioral skills model to understand PrEP intentions and use among men who have sex with men. AIDS Behav.

[34] Lenth RV (2001). Some practical guidelines for effective sample size determination. Am Stat.

[35] Whitehead AL, Julious SA, Cooper CL, Campbell MJ (2016). Estimating the sample size for a pilot randomised trial to minimise the overall trial sample size for the external pilot and main trial for a continuous outcome variable. Stat Methods Med Res.

[36] Julious SA (2005). Sample size of 12 per group rule of thumb for a pilot study. Pharm Stat.

[37] Sharma A, Lacombe-Duncan A, Sallabank G (2023). HIV and STI testing among South Asian gay, bisexual, and other men who have sex with men in the United States. Am J Mens Health.

[38] Dai M, Grant Harrington N (2021). Understanding beliefs, intention, and behavior on daily PrEP uptake among MSM in California and New York. AIDS Educ Prev.

[39] Dai M, Harrington NG (2021). Intention to behavior: using the integrative model of behavioral prediction to understand actual control of PrEP uptake among gay men. Arch Sex Behav.

[40] Shrader CH, Craker L, Johnson AL (2024). Peer influence on motivation to use pre-exposure prophylaxis among Latino sexual minority men in Miami, Florida: a network autocorrelation model. AIDS Patient Care STDS.

[41] Harkness A, Lozano A, Bainter S (2023). Engaging Latino sexual minority men in PrEP and behavioral health care: multilevel barriers, facilitators, and potential implementation strategies. J Behav Med.

[42] Kelly JA, Walsh JL, DiFranceisco WJ (2024). Factors associated with PrEP use in a community sample of African American men who have sex with men (MSM) and transgender women (TGW) in the United States Midwest. AIDS Care.

[43] (2024). HIV infection risk, prevention, and testing behaviors among men who have sex with men—National HIV Behavioral Surveillance, 19 U.S. cities, 2023. https://stacks.cdc.gov/view/cdc/162349.

[44] Stephenson R, Chavanduka TMD, Sullivan S, Mitchell JW (2022). Partner support and communication for pre-exposure prophylaxis (PrEP) use among male couples. Arch Sex Behav.

[45] Gusakova S, Chin K, Ascigil E (2021). Communication patterns among male couples with open and monogamous agreements. Arch Sex Behav.

[46] Shover CL, DeVost MA, Cunningham NJ (2020). Structural, dosing, and risk change factors affecting discontinuation of pre-exposure prophylaxis (PrEP) in a large urban clinic. AIDS Educ Prev.

[47] Quinn KG, Zarwell M, John SA, Christenson E, Walsh JL (2020). Perceptions of PrEP use within primary relationships among young Black gay, bisexual, and other men who have sex with men. Arch Sex Behav.

[48] Goodreau SM, Carnegie NB, Vittinghoff E (2012). What drives the US and Peruvian HIV epidemics in men who have sex with men (MSM)?. PLOS One.

[49] Sullivan PS, Salazar L, Buchbinder S, Sanchez TH (2009). Estimating the proportion of HIV transmissions from main sex partners among men who have sex with men in five US cities. AIDS.

[50] Shaver J, Freeland R, Goldenberg T, Stephenson R (2018). Gay and bisexual men’s perceptions of HIV risk in various relationships. Am J Mens Health.

[51] Stephenson R, White D, Darbes L, Hoff C, Sullivan P (2015). HIV testing behaviors and perceptions of risk of HIV infection among MSM with main partners. AIDS Behav.

[52] Sharma A, Kahle E, Sullivan S, Stephenson R (2020). Relationship characteristics associated with perceptions of partners’ HIV testing behavior among male couples. AIDS Behav.

[53] Mitchell JW, Petroll AE (2012). Patterns of HIV and sexually transmitted infection testing among men who have sex with men couples in the United States. Sex Transm Dis.

[54] Kota KK, Mansergh G, Stephenson R, Hirshfield S, Sullivan P (2021). Sociodemographic characteristics of HIV pre-exposure prophylaxis use and reasons for nonuse among gay, bisexual, and other men who have sex with men from three US cities. AIDS Patient Care STDS.

[55] Mansergh G, Kota KK, Carnes N, Gelaude D (2023). Brief report: refusal of daily oral PrEP: implementation considerations and reported likelihood of using various HIV prophylaxis products in a diverse sample of MSM. J Acquir Immune Defic Syndr.

[56] Hill A, Hughes SL, Gotham D, Pozniak AL (2018). Tenofovir alafenamide versus tenofovir disoproxil fumarate: is there a true difference in efficacy and safety?. J Virus Erad.

[57] Tang EC, Vittinghoff E, Anderson PL (2018). Changes in kidney function associated with daily Tenofovir Disoproxil Fumarate/Emtricitabine for HIV preexposure prophylaxis use in the United States Demonstration Project. J Acquir Immune Defic Syndr.

[58] Mulligan K, Glidden DV, Anderson PL (2015). Effects of Emtricitabine/Tenofovir on bone mineral density in HIV-negative persons in a randomized, double-blind, placebo-controlled trial. Clin Infect Dis.

[59] Kauppinen KJ, Kivelä P, Sutinen J (2019). Switching from Tenofovir Disoproxil Fumarate to Tenofovir Alafenamide significantly worsens the lipid profile in a real-world setting. AIDS Patient Care STDS.

[60] Wohl DA, Spinner CD, Flamm J (2024). HIV-1 infection kinetics, drug resistance, and long-term safety of pre-exposure prophylaxis with emtricitabine plus tenofovir alafenamide (DISCOVER): week 144 open-label extension of a randomised, controlled, phase 3 trial. Lancet HIV.

[61] Pilkington V, Hill A, Hughes S, Nwokolo N, Pozniak A (2018). How safe is TDF/FTC as PrEP? A systematic review and meta-analysis of the risk of adverse events in 13 randomised trials of PrEP. J Virus Erad.

[62] Khosla N, Hahn L, Tran C (2024). US South Asian youths’ perspectives on the use of Complementary and Alternative Medicine (CAM). J Racial Ethn Health Disparities.

[63] Misra R, Balagopal P, Klatt M, Geraghty M (2010). Complementary and alternative medicine use among Asian Indians in the United States: a national study. J Altern Complement Med.

[64] Gómez W (2023). Assessing PrEP messaging and communication: a review of the qualitative literature. Curr Opin Psychol.

[65] Sullivan P, Brixner D, Lam JT, Hsiao A (2024). Overcoming barriers to HIV prevention: population health considerations on optimizing PrEP access. Am J Manag Care.

[66] Srikanth K, Killelea A, Strumpf A, Corbin-Gutierrez E, Horn T, McManus KA (2022). Associated costs are a barrier to HIV preexposure prophylaxis access in the United States. Am J Public Health.

[67] Haines M, Vandyk A, Skidmore B, Orser L, O’Byrne P (2024). A systematic review of oral pre-exposure prophylaxis HIV adherence interventions. J Assoc Nurses AIDS Care.

[68] Garcia C, Rehman N, Matos-Silva J (2024). Interventions to improve adherence to oral pre-exposure prophylaxis: a systematic review and network meta-analysis. AIDS Behav.

[69] Meyers K, Wu Y, Brill A, Sandfort T, Golub SA (2018). To switch or not to switch: intentions to switch to injectable PrEP among gay and bisexual men with at least twelve months oral PrEP experience. PLOS One.

[70] Bonett S, Li Q, Sweeney A, Gaither-Hardy D, Safa H (2024). Telehealth models for PrEP delivery: a systematic review of acceptability, implementation, and impact on the PrEP care continuum in the United States. AIDS Behav.

[71] Rutstein SE, Muessig KE (2024). Leveling Up PrEP: implementation strategies at system and structural levels to expand PrEP use in the United States. Curr HIV/AIDS Rep.

[72] Raza F, Sakamoto A (2024). Socioeconomic attainments of second-generation South Asian Americans: evidence from the American Community Survey, 2014–2018. Popul Res Policy Rev.

