# Supplementary material for: HIV Pre-Exposure Prophylaxis Use Among South Asian Sexual Minority Men in the United States: Pilot Web-Based Survey Study
Source: JMIR Form Res. 2026 Aug 11;10:e88920. doi: 10.2196/88920 (PMC13460798; doi:10.2196/88920)
Supplement: Supplementary file 1 [file formative-v10-e88920-s001.pdf]

Section A. Landing Page

Thanks for taking the time to check out Ujala! This University of Michigan research study seeks to understand HIV-related knowledge, attitudes, and behaviors among South Asian gay, bisexual, and other men who have sex with men living in the US.

What's involved in participating?

1. Review the informed consent form and decide whether you are willing to volunteer for our research study.

2. Complete a short online eligibility screener that should take about 2 minutes and continue to a longer survey if you are eligible.

3. Complete a longer online survey that should take about 45 minutes and receive a $25 Amazon e-gift card after finishing.

Yes, that's about it! If you're interested in learning more, please click on the "Find out More" button to see the informed consent form. Thanks again!

Section B. Eligibility Screener

A. Do you currently identify as male?
   - Yes
   - No*

B. Are you 18 years of age or older?
   - Yes
   - No*

C. Do you currently live in a US state or territory?
   - Yes
   - No*

D. Are you of legal age to consent for yourself in the US state or territory in which you live?
   - Yes
   - No*

E. Do you identify as South Asian?
   - Yes

- No*

F. How many male sex partners have you had in the past 6 months?
    - None*
    - 1
    - 2 or more

G. Have you ever been diagnosed with HIV?
    - Yes*
    - No

H. Are you willing to provide a name and email address at the end of the survey to receive a $25 Amazon e-gift card?
    - Yes
    - No*

[If any answers marked with an asterisk (*) are selected, the individual is not eligible to participate. Otherwise, the individual is eligible to participate.]

[Individuals who are not eligible are shown the following text.]

Thank you for completing the eligibility screener!

Unfortunately, you are not eligible to participate in our research study at this time because you do not meet some of our criteria.

Please click on the link below for information and resources on HIV prevention. Otherwise, you may simply close your browser to exit.

[CDC's HIV Risk Reduction Tool Link]

[Individuals who are eligible are shown the following text.]

Thank you for completing the eligibility screener!

Based on your answers, you are eligible to participate in our research study.

Please click on the "Continue" button below to start the survey. Remember that all your answers are confidential, so please answer honestly. You can choose to skip any questions you do not feel comfortable answering for any reason. Once you have completed 80% of the survey, you will be eligible to receive a $25 Amazon e-gift card!

Section C. Main Survey

1. How did you hear about our research study?
    - Facebook advertisement
    - Facebook post
    - Instagram advertisement
    - Instagram post
    - Through a friend or a sex partner

Category I – Sociodemographic characteristics

2. What is your age?
    - Drop down list of ages ranging from 18-99

3. How would you describe your ethnic origin?
    - Bangladeshi
    - Bhutanese
    - Indian
    - Maldivian
    - Nepali
    - Pakistani
    - Sri Lankan
    - Not listed, please specify: __________

4. In which state or US territory do you currently live?
    - Drop down list of states and US territories

5. What is your current Zip Code?
    - __________

6. Were you born in the US?
    - Yes
    - No

7. For how long have you lived in the US?
    - Since birth
    - Less than 1 year

- 1 to 5 years
- 6-10 years
- More than 10 years

8. What is your present religion, if any?
   - Atheism (lack of belief in any gods)
   - Agnosticism (uncertainty about the existence of any gods)
   - Buddhism
   - Christianity
   - Hinduism
   - Islam
   - Jainism
   - Sikhism
   - Nothing in particular
   - Not listed, please specify: ___________

9. What is the highest level of education you have completed?
   - Some high school or less
   - High school diploma or GED
   - Some college (no degree)
   - Associate’s or Technical degree
   - Bachelor’s degree
   - Master’s or Doctoral degree

10. What is your current employment status?
    - Work full-time (30+ hours per week)
    - Work part-time (1-29 hours per week)
    - Student (full-time or part-time)
    - Not currently employed
    - Collecting disability
    - Retired
    - Not listed, please specify: ___________

11. What kind of health insurance do you currently have?
    - Private health plan (through an employer or school or purchased directly)
    - Medicaid or Medicare
    - Affordable Care Act (ACA)
    - COBRA
    - Veterans Affairs (VA) healthcare
    - Do not currently have health insurance
    - Not listed, please specify: ___________

12. Where do you usually receive healthcare services?
    - Private doctor's office
    - Community health center
    - Urgent care center or walk-in clinic
    - Hospital emergency room
    - School or college clinic
    - Do not have a usual source of healthcare
    - Not listed, please specify: ___________

13. What is your sexual orientation?
    - Gay
    - Bisexual
    - Straight
    - Queer
    - Questioning
    - Asexual
    - Pansexual
    - Not listed, please specify: ___________

Category II – Behaviors

14. Have you ever been tested for HIV?
    - Yes
    - No

[Answer Questions 15, 16, 17 and 18 if Question 14 = "Yes"]

15. How often do you get tested for HIV?
    - Every 3 months
    - Every 6 months
    - Every year
    - Every time I think I might have been exposed
    - I do not test regularly

16. When did you take your most recent HIV test?
    - In the past 3 months
    - 3 months to 6 months ago
    - 6 months to 1 year ago

- 1 to 2 years ago
- More than 2 years ago

17. Where did you take your most recent HIV test?
    - Private doctor's office
    - Community health center
    - Urgent care center or walk-in clinic
    - Hospital emergency room
    - School or college clinic
    - HIV or other sexually transmitted infection testing center
    - Mobile testing unit
    - Home or other private location
    - Not listed, please specify: __________

18. Why did you take your most recent HIV test?
    - A healthcare provider recommended I get tested
    - A sex partner requested I get tested
    - A friend encouraged me to get tested
    - I get tested regularly and it was time
    - I started a new relationship
    - I had unprotected sex
    - I had sex with someone who was HIV-positive
    - I had another sexually transmitted infection
    - I shared needles or syringes with someone
    - To start or continue taking PrEP
    - Testing was free at an outreach or community event
    - Not listed, please specify: __________

[Answer Question 19 if Question 14 = "No"]

19. Why have you never been tested for HIV? Select all that apply.
    - I have not had the time
    - I do not think I am at risk for HIV
    - I cannot afford getting tested for HIV
    - I am unsure if my insurance covers HIV testing
    - I feel uncomfortable asking my healthcare provider about HIV testing
    - I am afraid of finding out my HIV test result
    - I am worried that someone else will find out my HIV test result
    - I do not know where to get tested for HIV
    - I do not have transportation to an HIV testing location
    - Not listed, please specify: __________

20. Have you heard about HIV self-testing kits that you can buy at a pharmacy or online and use on your own at home or in a private location (e.g., OraQuick In-Home HIV Test, INSTI HIV Self Test)?
    - Yes
    - No

21. Have you ever been tested for other sexually transmitted infections (e.g., gonorrhea, chlamydia, syphilis)?
    - Yes
    - No

[Answer Questions 22, 23, and 24 if Question 21 = "Yes"]

22. How often do you get tested for other sexually transmitted infections (e.g., gonorrhea, chlamydia, syphilis)?
    - Every 3 months
    - Every 6 months
    - Every year
    - Every time I think I might have been exposed
    - I do not test regularly

23. When did you take your most recent test for other sexually transmitted infections (e.g., gonorrhea, chlamydia, syphilis)
    - In the past 3 months
    - 3 months to 6 months ago
    - 6 months to 1 year ago
    - 1 to 2 years ago
    - More than 2 years ago

24. Where did you take your most recent test for other sexually transmitted infections (e.g., gonorrhea, chlamydia, syphilis)?
    - Private doctor's office
    - Community health center
    - Urgent care center or walk-in clinic
    - Hospital emergency room
    - School or college clinic
    - HIV or other sexually transmitted infection testing center
    - Mobile testing unit

- Home or other private location
- Not listed, please specify: __________

25. Are you currently in a committed relationship with another man? You might call this person your boyfriend, partner, husband, spouse or significant other.
    - Yes
    - No

[Answer Question 26 if Question 25 = “Yes”]

26. Do you and this person have an agreement about whether or not you can have sex with partners outside your relationship?
    - Yes
    - No

[Answer Question 27 if Question 26 = “Yes”]

27. What is the agreement that you and this person have about having sex with partners outside your relationship?
    - Neither of us can have sex with outside partners
    - Both of us can have sex with outside partners, without any conditions or restrictions
    - Both of us can have sex with outside partners, but with some conditions or restrictions

28. How many men have you had anal sex with in the past 6 months? By anal sex we mean “topping” or “bottoming”?
    - None
    - 1 (someone I am in a committed relationship with)
    - 1 (someone I am not in a committed relationship with)
    - 2
    - 3 or more

[Answer Questions 29 and 30 if Question 28 = “1 (someone I am in a committed relationship with)” or “1 (someone I am not in a committed relationship with)”]

29. Did you use a condom the entire time you had anal sex with this partner?

- Yes
- No

30. What was this sex partner's HIV status?
    - Positive
    - Negative
    - I don't know

[Answer Questions 31 and 32 if Question 28 = "2" or "3 or more"]

31. Did you use a condom the entire time you had anal sex with these partners?
    - Yes
    - No

32. Were any of these sex partners HIV-positive?
    - Yes
    - No
    - I don't know

33. Have you ever taken pre-exposure prophylaxis (PrEP) to reduce your risk of getting HIV?
    - Yes
    - No

[Answer Question 34 if Question 33 = "Yes"]

34. Are you currently taking pre-exposure prophylaxis (PrEP) to reduce your risk of getting HIV?
    - Yes
    - No

[Answer Question 35 if Question 34 = "Yes"]

35. Do you currently take pre-exposure prophylaxis (PrEP) every day, almost every day, or less often?
    - Every day

- Almost every day
- Less often

[Answer Question 36 if Question 34 = “No”]

36. Did you previously take pre-exposure prophylaxis (PrEP) every day, almost every day, or less often?
    - Every day
    - Almost every day
    - Less often

[Answer Question 37 if Question 34 = “No”]

37. Why did you stop taking pre-exposure prophylaxis (PrEP)? Select all that apply.
    - I could not afford it
    - My insurance would not cover it
    - I was having side effects
    - I was worried about its long-term safety
    - I started a committed relationship
    - I decided I did not need it anymore
    - Taking it was too inconvenient
    - My healthcare provider decided it was no longer right for me
    - I was worried someone would think I was HIV-positive if they saw me take it
    - Not listed, please specify: ___________

[Answer Question 38 if Question 33 = “No”]

38. Why have you never taken pre-exposure prophylaxis (PrEP)? Select all that apply.
    - I cannot afford it
    - My insurance does not cover it
    - I am worried about its side effects
    - I am worried about its long-term safety
    - I am in a committed relationship
    - I do not think I need it
    - Taking it is too inconvenient
    - My healthcare provider does not think it is right for me
    - I am worried someone would think I am HIV-positive if they saw me take it
    - I do not know where to get it
    - I do not think I am at risk for HIV

- Not listed, please specify: __________

39. Have you heard about companies that allow you to book virtual appointments with healthcare providers to determine whether pre-exposure prophylaxis (PrEP) is right for you and deliver prescriptions at home (e.g., PlushCare, NURX)?
    - Yes
    - No

40. In the past 6 months, how often did you use the following substances?

	Never	Once or Twice	Monthly	Weekly	Daily or Almost Daily
Alcohol (5 or more drinks a day)					
Tobacco products					
Cannabis (marijuana, pot)					
Inhalants (paint thinner, aerosol, glue, alkyl nitrites or “poppers”)					
Methamphetamines (speed, crystal)					
Hallucinogens (LSD, mushrooms, MDMA or Ecstasy/Molly)					
Tranquilizers (valium)					
Narcotics (heroin, oxycodone, methadone)					
Cocaine					

41. In the past 6 months, did you use any drugs that you injected with a needle, other than those prescribed for you by a healthcare provider?
    - Yes
    - No

42. In the past 6 months, did you use alcohol or drugs immediately before or during sex?
    - Yes
    - No

Category III – Information

43. The following items are designed to assess your knowledge of HIV transmission and prevention. For each statement, please select “True”, “False”, or “I don’t know”. If you do not know, please do not guess; instead, please select “I don’t know”.

	T	F	IDK
Coughing and sneezing do NOT spread HIV			
A person can get HIV by sharing a glass of water with someone who has HIV			
Pulling out the penis before a man climaxes (cums) keeps a person from getting HIV during sex			
A person can get HIV from having anal sex			
Showering or washing one's genitals (private parts) after sex keeps a person from getting HIV			
All pregnant women who are HIV-positive will have babies born with HIV			
People who have been infected with HIV quickly show serious signs of being infected			
There is a vaccine that can stop people from getting HIV			
People are likely to get HIV by deep kissing, putting their tongue in a partner's mouth, if their partner has HIV			
A woman cannot get HIV if she has sex during her period			
There is a female condom that can help decrease a woman's chance of getting HIV			
A natural skin or lamb skin condom works better against HIV than does a latex condom			
A person will NOT get HIV if they are taking antibiotics			
Having sex with more than one partner can increase a person's chance of being infected with HIV			
Taking a test for HIV one week after having sex will tell a person if they have HIV			
A person can get HIV by sitting in a hot tub or a swimming pool with a person who has HIV			
A person can get HIV from oral sex			
Using Vaseline or baby oil with condoms lowers the chance of getting HIV			

Note: Items in this question were adapted from "Carey, M. P., & Schroder, K. E. (2002). Development and psychometric evaluation of the brief HIV Knowledge Questionnaire. AIDS education and prevention, 14(2), 172-182."

44. The following items are designed to assess your knowledge of pre-exposure prophylaxis (PrEP). For each statement, please select "True", "False", or "I don't know". If you do not know, please do not guess; instead, please select "I don't know".

	T	F	IDK
PrEP is a daily pill you can take to reduce your risk of getting HIV			
You should not use PrEP if you don't know your HIV status			
If you do not take PrEP consistently, there may not be enough medicine in your bloodstream to block the HIV virus			
PrEP can be used to prevent STIs like gonorrhea, chlamydia, syphilis, herpes, and HPV			

Many insurance plans will cover PrEP			
If you start taking PrEP, you will have to take it for the rest of your life			
PrEP can be taken by people who already have HIV			
There are ways to get PrEP even if you do not have insurance			
PrEP is available over-the-counter			
You must take an HIV test every 3 months while taking PrEP			
There are many serious side effects of taking PrEP			
The PrEP pill contains two medicines that are also used to treat HIV			
Daily PrEP use can lower the risk of getting HIV from sex by more than 90%			

Note: Items in this question were adapted from "Walsh, J. L. (2019). Applying the information–motivation–behavioral skills model to understand PrEP intentions and use among men who have sex with men. AIDS and Behavior, 23(7), 1904-1916."

Category IV – Motivation

45. The following items are designed to assess your perceived risk of HIV infection. Please choose the response that best describes how you think and feel about your risk of HIV infection based on your sexual behaviors.

What is your gut feeling about how likely you are to get infected with HIV?	• Extremely unlikely<br>• Very unlikely<br>• Somewhat likely<br>• Very likely<br>• Extremely likely
I worry about getting infected with HIV	• None of the time<br>• Rarely<br>• Some of the time<br>• A moderate amount of time<br>• A lot of the time<br>• All of the time
Picturing self getting HIV is something I find:	• Very hard to do<br>• Hard to do<br>• Easy to do<br>• Very easy to do
I am sure I will NOT get infected with HIV	• Strongly disagree<br>• Disagree<br>• Somewhat disagree<br>• Somewhat agree<br>• Agree<br>• Strongly agree

I feel vulnerable to HIV infection	• Strongly disagree<br>• Disagree<br>• Somewhat disagree<br>• Somewhat agree<br>• Agree<br>• Strongly agree
There is a chance, no matter how small, I could get HIV	• Strongly disagree<br>• Disagree<br>• Somewhat disagree<br>• Somewhat agree<br>• Agree<br>• Strongly agree
I think my chances of getting infected with HIV are:	• Zero<br>• Almost zero<br>• Small<br>• Moderate<br>• Large<br>• Very Large
Getting HIV is something I have:	• Never thought about<br>• Rarely thought about<br>• Thought about some of the time<br>• Thought about often

Note: Items in this question were adapted from "Napper, L. E., Fisher, D. G., & Reynolds, G. L. (2012). Development of the perceived risk of HIV scale. AIDS and Behavior, 16(4), 1075-1083."

46. The following items are designed to assess your attitudes around HIV testing. For each statement, please select "Strongly agree", "Agree", "Neutral", "Disagree" or "Strongly disagree".

	SA	A	N	D	SD
My friends would support my decision to get an HIV test					
I could talk to my friends about making the decision to get an HIV test					
My family would support me if I decided to be tested for HIV					
It would not bother me if someone I know sees me going to get an HIV test					
I could easily discuss HIV antibody testing with my family					
My friends would not treat me any different if I were tested for HIV					
I am afraid that if I were to be tested for HIV, my name would go into public records					
I do not have time to get an HIV test					
Anyone who is tested for HIV is disgusting					
HIV antibody testing is not really confidential					

I would not consider getting an HIV test because I would be asked about things I have done that could get me into trouble					
I would be embarrassed if my friends found out I had decided to have an HIV test					
People would assume I have HIV if I decided to get tested					
I am afraid someone would find out I was tested for HIV					
I would not get tested for HIV because I would be asked information that was too personal					
My parents would be upset if they knew I was planning to get tested for HIV					
HIV antibody testing information is kept very confidential by the medical staff who do testing					
I trust the HIV counselors and nurses to keep my information confidential					
I can talk to my friends about making medical decisions					
My friends would look down on me if I were tested for HIV					
My friends would treat me badly if I were to be tested for HIV					
My friends would not look down on me if I were tested for HIV					

Note: Items in this question were adapted from “Peltzer, K., & Mpofu, E. (2002). The factor structure of the HIV antibody testing attitude scale in four African countries. Eastern Journal of Medicine, 7(2), 27-30.”

47. How likely are you to get tested for HIV in the next 12 months?
    - Extremely unlikely
    - Very unlikely
    - Somewhat likely
    - Very likely
    - Extremely likely

48. The following items are designed to assess your attitudes around using condoms. For each statement, please select “Strongly agree”, “Agree”, “Neutral”, “Disagree” or “Strongly disagree”.

	SA	A	N	D	SD
The proper use of a condom could enhance sexual pleasure					
Condoms ruin the natural sex act					
Condoms interfere with romance					
Generally, I am in favor of using condoms					
“Safer” sex reduces the mental pleasure of sex					
The idea of using a condom doesn’t appeal to me					

The sensory aspects (smell, touch, etc.) of condoms make them unpleasant					
With condoms, you can't really "give yourself over" to your partner					
I think "safer" sex would get boring fast					
Condoms are irritating					
People can get the same pleasure from "safer" sex as from unprotected sex					
Using condoms interrupts sex play					
It is a hassle to use condoms					
My friends talk a lot about "safer" sex					
My friends and I encourage each other before dates to practice "safer" sex					
When I think that one of my friends might have sex on a date, I ask them if they have a condom					
If I thought that one of my friends had sex on a date, I would ask them if they used a condom					
If a friend knew that I might have sex on a date, they would ask me if I were carrying a condom					
If a friend knew that I had sex on a date, they wouldn't care if I had used a condom or not					
If I had sex and I told my friends that I did not use condoms, they would be angry or disappointed					

Note: Items in this question were adapted from "DeHart, D. D., & Birkimer, J. C. (1997). Trying to practice safer sex: Development of the sexual risks scale. Journal of Sex Research, 34(1), 11-25."

49. If you were having anal sex with a partner in the next 12 months, how likely would you be to use a condom (if you were the "top" partner) or persuade your partner to use a condom (if you were the "bottom" partner)?
    - Extremely unlikely
    - Very unlikely
    - Somewhat likely
    - Very likely
    - Extremely likely

50. The following items are designed to assess your attitudes around using pre-exposure prophylaxis (PrEP). For each statement, please select "Strongly agree", "Agree", "Neutral", "Disagree" or "Strongly disagree".

	SA	A	N	D	SD
PrEP is effective at preventing HIV					
People who take PrEP are responsible					
Taking PrEP is safe					
It would be no trouble to take PrEP every day					

The government makes certain that drugs like PrEP are safe					
People who take PrEP are promiscuous					
Getting a PrEP prescription from a doctor would be embarrassing					
If I were to take PrEP, I would be concerned if my friends found out I was taking it					
If I were to take PrEP, I would be concerned if my family found out I was taking it					
If I were to take PrEP, I would be concerned if my sexual partner(s) found out I was taking it					
My friends would be supportive of me using PrEP					
My friends would think it was smart if I used PrEP					
My friends would think it was responsible if I used PrEP					
My sexual partner(s) would be supportive of me using PrEP					
My sexual partner(s) would think it was smart if I used PrEP					
My sexual partner(s) would think it was responsible if I used PrEP					
People in my community would be interested in learning more about PrEP					
People in my community would be willing to talk with their doctors about using PrEP					
People in my community would consider taking PrEP					
My friends would be interested in learning more about PrEP					
My friends would be willing to talk with their doctors about using PrEP					
My friends would consider taking PrEP					

Note: Items in this question were adapted from "Walsh, J. L. (2019). Applying the information–motivation–behavioral skills model to understand PrEP intentions and use among men who have sex with men. AIDS and Behavior, 23(7), 1904-1916."

51. If a healthcare provider were to prescribe pre-exposure prophylaxis (PrEP) to you in the next 12 months based on your pattern of sexual risk, how likely would you be to use PrEP to prevent HIV?
    - Extremely unlikely
    - Very unlikely
    - Somewhat likely
    - Very likely
    - Extremely likely

Category V – Behavioral Skills

52. The following items are designed to assess your confidence around HIV testing. For each statement, please select “Not at all confident”, “Not very confident”, “Somewhat confident”, “Very confident” or “Completely confident”.

	NAC	NVC	SC	VC	CC
Getting tested for HIV at least once per year					
Getting an HIV test after unsafe sex					
Asking my doctor or nurse for an HIV test					
Discussing unsafe sex with a doctor or nurse					
Buying a home HIV test from a chemist					
Using a home HIV test myself					
Understanding the results of a home HIV test					
Knowing how long to wait after unsafe sex before getting tested for HIV					

Note: Items in this question were adapted from “Jamil, M. S., Guy, R. J., Bavinton, B. R., Fairley, C. K., Grulich, A. E., Holt, M., ... & FORTH Investigator Group. (2016). HIV testing self-efficacy is associated with higher HIV testing frequency and perceived likelihood to self-test among gay and bisexual men. Sexual health, 14(2), 170-178.”

53. The following items are designed to assess your confidence around using condoms. For each statement, please select “Strongly agree”, “Agree”, “Neutral”, “Disagree” or “Strongly disagree”.

	SA	A	N	D	SD
I feel confident in my ability to put a condom on myself or my partner					
I feel confident in my ability to use a condom correctly					
I feel confident in my ability to put a condom on myself or my partner quickly					
If I were to suggest using a condom to a partner, I would feel afraid that they would reject me					
If I were unsure of my partner’s feelings about using condoms, I would not suggest using one					
I would not feel confident suggesting using condoms with a new partner because I would be afraid they would think I’ve had a past gay experience					
I would not feel confident suggesting using condoms with a new partner because I would be afraid they would think I have a sexually transmitted disease					
I would not feel confident suggesting using condoms with a new partner because I would be afraid they would think I thought they had a sexually transmitted disease					
I feel confident in my ability to discuss condom usage with any partner I might have					
I feel confident in my ability to suggest using a condom with a new partner					
I feel confident that I could suggest using a condom without my partner feeling “diseased”					

I feel confident that I would remember to use a condom even after I have been drinking					
I feel confident that I would remember to use a condom even if I were high					
I feel confident I could stop to put a condom on myself or my partner even in the heat of passion					

Note: Items in this question were adapted from "Brien, T. M., Thombs, D. L., Mahoney, C. A., & Wallnau, L. (1994). Dimensions of self-efficacy among three distinct groups of condom users. Journal of American College Health, 42(4), 167-174."

54. The following items are designed to assess your confidence around using pre-exposure prophylaxis (PrEP). For each statement, please select if it would be "Very hard to do", "Somewhat hard to do", "Somewhat easy to do", or "Very easy to do".

	VH	SH	SE	VE
Seek out more information about PrEP to decide if it is right for you				
Talk with your sexual partner(s) about the decision to take PrEP				
Visit a doctor who can provide PrEP				
Talk openly and honestly with a doctor about your sexual behaviors				
Get tested for HIV				
Find a way to pay for PrEP				
Take a medicine like PrEP every day				
Visit a doctor every 3 months for routine screenings				

Note: Items in this question were adapted from "Walsh, J. L. (2019). Applying the information–motivation–behavioral skills model to understand PrEP intentions and use among men who have sex with men. AIDS and Behavior, 23(7), 1904-1916."

Category VI – Minority stressors

55. What percent of the people in this group do you think are aware of your sexual orientation (meaning they are aware of whether you consider yourself gay, bisexual, straight, etc.)?

	0 %	10 %	20 %	30 %	40 %	50 %	60 %	70 %	80 %	90 %	100 %
Members of your immediate family (e.g., parents and siblings)											
Members of your extended family (e.g., aunts, uncles, grandparents, cousins)											

People you socialize with (e.g., friends and acquaintances)											
People at your work or school (e.g., coworkers, supervisors, instructors, students)											
Strangers (e.g., someone you have a casual conversation with in line at the store)											

Note: Items in this question were adapted from "Meidlinger, P. C., & Hope, D. A. (2014). Differentiating disclosure and concealment in measurement of outness for sexual minorities: The Nebraska Outness Scale. Psychology of Sexual Orientation and Gender Diversity, 1(4), 489."

56. How often do you avoid talking about topics related to or otherwise indicating your sexual orientation (e.g., not talking about your significant other, changing your mannerisms) when interacting with members of these groups?

	Never					Half of the time					Always
Members of your immediate family (e.g., parents and siblings)											
Members of your extended family (e.g., aunts, uncles, grandparents, cousins)											
People you socialize with (e.g., friends and acquaintances)											
People at your work or school (e.g., coworkers, supervisors, instructors, students)											
Strangers (e.g., someone you have a casual conversation with in line at the store)											

Note: Items in this question were adapted from "Meidlinger, P. C., & Hope, D. A. (2014). Differentiating disclosure and concealment in measurement of outness for sexual minorities: The Nebraska Outness Scale. Psychology of Sexual Orientation and Gender Diversity, 1(4), 489."

57. The following items are designed to assess your experiences with homophobia. For each statement, please select how often these things happened to you from "Never", "Rarely", "Sometimes", or "Often".

	N	R	S	O
While growing up, how often …				
did your family make fun of you or call you names because of the way you behaved?				

did other kids make fun of you or call you names because of the way you behaved?				
did members of your family tell you to change your behavior because you were too effeminate (“girly”)?				
were you pushed around or beaten up because of the way you behaved?				
As an adult, how often …				
has your family made fun of you or called you names because of your sexual orientation?				
have other people made fun of you or called you names because of your sexual orientation?				
have you been rejected by your family because of your sexual orientation?				
have you been rejected by your friends because of your sexual orientation?				
have you moved away from your family and friends because of your sexual orientation?				
have you been told to consult a mental health professional because of your sexual orientation?				
have you been treated unfairly at work because of your sexual orientation?				
have you been treated differently in social situations because of your sexual orientation?				
have you received poor service because of your sexual orientation?				
have you been pushed around or beaten up because of your sexual orientation?				

Note: Items in this question were adapted from “Ramirez-Valles, J., Kuhns, L. M., Campbell, R. T., & Diaz, R. M. (2010). Social integration and health: Community involvement, stigmatized identities, and sexual risk in Latino sexual minorities. Journal of Health and Social Behavior, 51(1), 30-47.”

58. The following items focus on your cultural identity (South Asian) and your sexual orientation (meaning whether you consider yourself gay, bisexual, straight, etc.) For each statement, please select “Strongly agree”, “Agree”, “Somewhat agree”, “Neutral”, “Somewhat disagree”, “Disagree” or “Strongly disagree”.

	SA	A	SA	N	SD	D	SD
I have personally experienced cultural prejudice within the lesbian, gay, and bisexual community							
I am angry at the way the lesbian, gay, and bisexual community treats members of my cultural group (South Asians)							
I have found the lesbian, gay, and bisexual community to be embracing of my South Asian identity							

I have felt rejected by the lesbian, gay, and bisexual community because of my South Asian identity							

Note: Items in this question were adapted from “Sarno, E. L., Mohr, J. J., Jackson, S. D., & Fassinger, R. E. (2015). When identities collide: Conflicts in allegiances among LGB people of color. Cultural Diversity & Ethnic Minority Psychology, 21(4), 550.”

[Participants who reach the end of the survey are shown the following text.]

Thanks for participating in our research study!

Please click on the link below for information and resources on HIV prevention.

[CDC’s HIV Risk Reduction Tool Link]

If you have completed 80% of the survey, you are eligible to receive a $25 Amazon e-gift card. Please click on the “Continue” button below to give us your name and email address at which you would like to receive this e-gift card. In order to protect your identity, your name and email address will be stored separately from your survey responses.

Section D. Contact Information Form

Name: ____________________

Email address: ____________________

Thanks for providing your name and email address! One of our study team members will get in touch with you shortly and ask you to reply to a test email to confirm its accuracy and functionality before sending you a $25 Amazon e-gift card.

If you know anyone who might be interested in participating, please copy the link below and share it with them. Thanks again!

[Landing Page Link]
